# Shared mental representations underlie metaphorical sound concepts

**DOI:** 10.1038/s41598-023-32214-2

**Published:** 2023-03-30

**Authors:** Victor Rosi, Pablo Arias Sarah, Olivier Houix, Nicolas Misdariis, Patrick Susini

**Affiliations:** 1 Sound Perception and Design Group, STMS, Ircam − Sorbonne Université − CNRS − Ministère de la Culture, 1 Place Igor Stravinsky, 75004 Paris, France; 2 School of Psychology and Neuroscience, University of Glasgow, 62 Hillhead Street, Glasgow, G12 8QB UK; 3 Lund University Cognitive Science, Lund University, Box 192, 221 00 Lund, Sweden

**Keywords:** Psychology, Human behaviour

## Abstract

Communication between sound and music experts is based on the shared understanding of a metaphorical vocabulary derived from other sensory modalities. Yet, the impact of sound expertise on the mental representation of these sound concepts remains blurry. To address this issue, we investigated the acoustic portraits of four metaphorical sound concepts (brightness, warmth, roundness, and roughness) in three groups of participants (sound engineers, conductors, and non-experts). Participants (N = 24) rated a corpus of orchestral instrument sounds (N = 520) using Best–Worst Scaling. With this data-driven method, we sorted the sound corpus for each concept and population. We compared the population ratings and ran machine learning algorithms to unveil the acoustic portraits of each concept. Overall, the results revealed that sound engineers were the most consistent. We found that roughness is widely shared while brightness is expertise dependent. The frequent use of brightness by expert populations suggests that its meaning got specified through sound expertise. As for roundness and warmth, it seems that the importance of pitch and noise in their acoustic definition is the key to distinguishing them. These results provide crucial information on the mental representations of a metaphorical vocabulary of sound and whether it is shared or refined by sound expertise.

## Introduction

Regardless of the field, human experts need a precise technical vocabulary to accurately communicate with each other. In some professional areas, such as perfumery, oenology^1,2^, or music^3^, experts often use metaphorical concepts from other modalities to describe their sensory experiences^4,5^. For instance, sound and music experts such as sound engineers, musicians, or sound designers employ terms from the senses of vision and touch like “bright”, “harsh”, “rough” or “sharp” to describe sounds^3,6,7^. However, because of their metaphorical nature, the mental representations associated with such concepts remain vastly unexplored and are not guaranteed to be identical between individuals with different sound professional activity and training backgrounds. In this study, we investigate whether the mental representations associated with metaphorical sound concepts are shared between populations.

Verbally describing sound properties is a key aspect of professional communication for music and sound professionals. It can happen when the conductor gives a stylistic comment to the principal oboist of an orchestra: “Could you please play this melody brighter?” during the conversation between this very same conductor and a sound engineer during the mixing session of a recording: “The mix should highlight the warmth of the cello section, here”; or between a sound designer and a marketing executive that has no sound expertise when designing a human–computer interface. Previous studies have consistently observed and analyzed the use of metaphorical sound concepts in the discourse of professionals in different languages of the Western world^3,7–10^. Such studies highlight that metaphorical sound concepts are mostly used to describe a multidimensional aspect of sound known as timbre. Timbre is used by listeners to distinguish from a wide range of sound sources—from musical instruments^11^ to everyday sounds^12^. From an acoustic perspective, timbre perception studies provided estimations of perceptual dimensions of timbre (such as instruments) using spectral, temporal, and spectro-temporal features. In this line, metaphorical concepts have been linked to timbral acoustic features^11,13–15^. Naturally, numerous subsequent studies intended to establish links between perceptual dimensions, sound semantics, and acoustic features. For example, brightness is often linked to the spectral centroid^10,16,17^.

However, recent studies also reported that the meaning of some well-known metaphorical sound concepts are highly dependent on each other^3,10,18^. For instance, the concept of 'warmth', or ’roundness' have really similar and intermingled meanings^10,19^ to the point of using one to define the other^18^—making them even more difficult to be explicit on their own. Similarly, the concepts of ‘brightness’, ‘sharpness’, and ‘clarity’ are very close^18,20^ while potentially being used for different purpose^20^. Furthermore, the relations of opposition existing between multiple concepts are not explicit. For instance, one may wonder if the observed opposition between roundness and brightness^18,19^ is dependent on comparable acoustic traits as the one between warmth and brightness^18,21^. Incidentally, it adds another degree of complexity to the definition and purpose of metaphorical sound concepts. In short, accurately communicating about sound with metaphorical concepts is not trivial.

From a cognitive perspective, accurate communication requires that individuals share a common mental representation associated with sound concepts. Such mental representations may develop from explicit pedagogical learning, cross-modal associations, or exposition to word-sound examples in professional contexts^22^. In consequence, different populations may develop different mental representations^23^. In other words, when two individuals with different professional backgrounds interact, they may be talking about different concepts, despite using exactly the same word. However, while previous studies have substantially investigated the influence of sound expertise on sound perception tasks^11,24–27^, it remains largely unknown whether the mental representations of metaphorical sound concepts are influenced by expertise or vary between expert populations.

**Figure 1:**
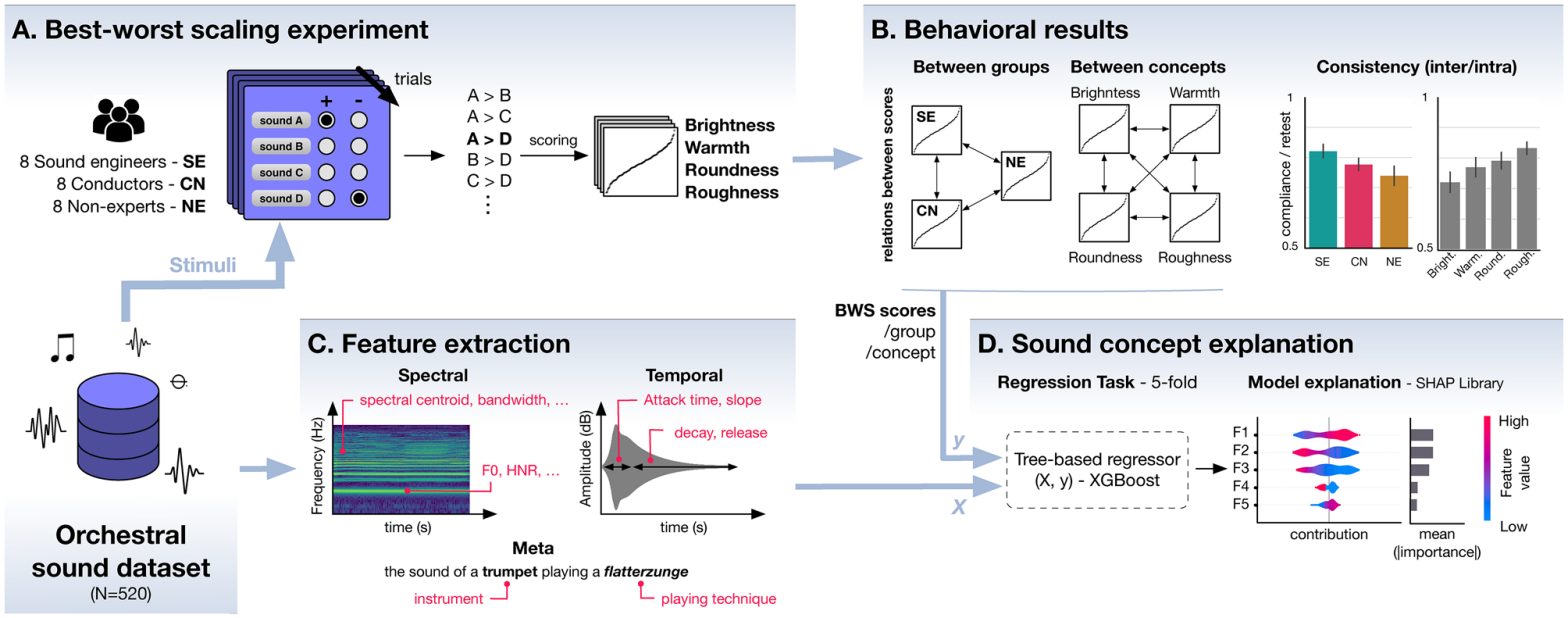
Schematic view of the methodology used to investigate the mental representations associated with specific sound concepts for different populations. (**A**) We collected ratings on an orchestral sound dataset using four sound concepts from three participant groups with the Best–Worst Scaling methods. (**B**) Using these ratings, we computed consistency metrics and measured similarities and differences between groups and between concepts. (**C**) We extracted acoustic features (e.g., spectral centroid, attack slope) and meta features (i.e., instrument, playing technique) from the sound dataset. (**D**) We trained a tree-based model and assessed the most important features for the prediction of the model.

In the present study, we investigate whether the mental representations of well-known sound concepts, i.e., brightness, warmth, roundness, and roughness, are similar between groups of participants with different sound education backgrounds. For this purpose, three groups of participants, namely, sound engineers, conductors, and non-experts evaluated a musical instrument sound corpus (N = 520) on brightness, warmth, roundness, and roughness. We chose these metaphorical concepts because of their metaphorical nature as they can be used to describe other sensory stimulations. Furthermore, they are frequently used in the professional fields of sound and music^3,6,7,28^, and show both strong similarities (e.g., roundness vs. warmth) and specificities (e.g., brightness/warmth, roundness/roughness)^18^ that we aim to investigate. The three participant groups display intrinsic homogeneity in terms of expertise. The sound engineers have a rather technical knowledge of sound, whereas conductors have an intertwined knowledge of music and sound. Both populations, however, are accustomed to the use of sound concepts, unlike the non-expert group, who reported a basic metaphorical use of these concepts that is not influenced by sound or music education. As part of an experiment, participants labelled a dataset of sounds with each sound concept using Best–Worst Scaling (BWS), a method based on sound comparisons that has shown good performance in measuring perceptual sound qualities. Subsequently, participants indicated how frequently they use said concept to talk about sounds in their professional life. Through the analysis of the consistency of judgments and acoustic modeling of BWS scores, we show the influence of the groups’ sound expertise on their shared understanding of those sound concepts. Figure 1 provides a schematic overview of the study conducted.

## Methods

### Participants

Twenty-four volunteer participants (mean age = 33, age-range = 25–65) took part in the experiment. They were organized in three groups of eight participants of different expertise: professional sound engineers (mean age = 31, age-range = 25–33; seven men, one woman), professional conductors (mean age = 37, age-range = 30–60, eight men) and non-experts (mean age = 33, age-range = 25–65; four men, four woman). The number of participants corresponds to the sufficient number of evaluations of the dataset that provides robust and consistent BWS scores according to the reference studies^29,30^. The non-expert group only included participants who reported no amateur nor professional practice related to sound or music (less than 2 years of music practice). All participants reported normal hearing and had no history of audiological or neurological disorders. The protocol (ID: 2021-76) was approved according to Helsinki Declaration by the Ethics Committee of *Institut Européen d’Administration des Affaires* (INSEAD). All methods were carried out in accordance with their guidelines and regulations. Participants gave written informed consent and received financial compensation for their participation.

### Setup

Sounds were presented to listeners diotically through a Beyerdynamic DT-770 PRO headset (80 Ohm) at an average level of 65 dB SPL. The sound level was measured with the sound level meter type 2250-S of Brüel & Kjær. Participants were tested in a double-walled IAC sound-insulated booth. The test interface was coded with Max (v8) on a Mac Mini.

### Stimuli

The sound corpus consisted of 520 musical instrument sounds (i.e., strings, brass, woodwinds, and keyboards) from the Studio-Online library^31^ and VSL (https://www.vsl.co.at). As in a previous study^18^, the sounds were selected arbitrarily on the basis of source, playing technique, variety of dynamics, and registers. Specifically, we retained 22 instruments with different playing techniques, e.g., *sul ponticello*, multiphonics, *flatterzunge*. Thus, the dataset included 140 string instrument sounds, 172 woodwind sounds, 102 brass sounds, 67 keyboard sounds, 16 harp sounds, 9 guitar sounds and 14 accordion sounds (see Supplementary information for the full report of instruments and playing techniques). To ensure that the stimuli covered the full spectral range, while controlling for harmonic interactions, we selected instrumental samples playing over several octaves of Cs ranging from C1 (32.70 Hz) to C8 (4186.01 Hz) with different dynamics. The loudness of the sound samples was equalized following the EBU norm on loudness (R-128) with the *ffmpeg* library (https://pypi.org/project/ffmpeg-python/). The sounds of the dataset were 5.5 s long on average and were ranging from 0.5 s (i.e., a *staccato* sound of French horn) to 15 s (i.e., a sound of harp with a long resonance).

### Procedure

We used Best–Worst Scaling^32^ (BWS) to collect ratings on the sound corpus. BWS is a subjective annotation method based on a stimuli comparison format that showed great performance for the evaluation of perceptual sound qualities^33^. In the context of sound evaluation, a BWS procedure consists of presenting k-tuples of sounds (e.g., k = 4), and asking participants to choose the best and the worst sound depending on the studied concepts. Final scores for each sound are computed by counting the number of best and worst judgments. Recent works have adapted BWS for the annotation of a large corpus of items^29,34^. Specifically, by considering each trial as a tournament paradigm^29^, the information taken from a trial is not the choice of best and worst but all the relations between each sound. For instance when evaluating brightness, if a participant chooses A as the brightest sound and D as the least bright sound in a group of sounds [A, B, C, D], then, in addition to the deducted information that A > D, we also consider that A > B, A > C, B > D, and C > D. Crucially, this paradigm allows us to propagate the information between different sequences of trials using a scoring algorithm based on the Rescorla-Wagner model^29,35^, and hence, compute the scores for all the sounds. To maximize the information propagated for the calculation of scores, a pair of sounds can only be presented once. We optimized the number of participants for each group based on the number of evaluations necessary to obtain consistent BWS scores^29^.

Participants completed BWS procedures for the four concepts in a randomized order, in two sessions of two blocks—one block for one concept. For each concept, participants evaluated the entire set of sounds through 130 trials of four sounds, with the addition of 13 retest trials to assess intra-participant consistency (see “Data Analysis” section). At each trial, participants had to listen to the four sounds before choosing the best and worst sounds according to the concept studied. A break was offered at the midpoint of a block. While meeting the constraint of presenting a pair of sound only once in the overall experiment, the grouping of sounds in trials was randomized. The configuration of sounds in each trial and the sequence of trials were also randomized for each concept. At the end of each block, participants used a 7-point Likert scale to rate how often they used the concept to describe a sound in professional settings. The average duration of a block for the evaluation of one concept was 36 min.

### Data analysis

#### Analysis of behavioral data

To measure whether the mental representations of specific sound concepts are shared between populations, we computed compliance scores—an individual measure of inter-participant consistency. Specifically, compliance is the proportion of matching duels of sounds between participant choices and means scores computed with the BWS scoring algorithm. For instance, if a participant from the sound engineer group answered that *sound A* > *sound B* because he or she chose sound A as 'best' in the trial [A, B, C, D], then, that participant’s compliance will increase if the BWS score of sound A is indeed greater than the one of sound B for the sound engineer group. In other words, a consistent group will have a higher average compliance score than a less consistent group. Random responses from a participant in the experiment would result in a compliance score of 50%. We tested for the influence of the concept and the group of participants on compliance with two Kruskall–Wallis tests, because of the non-normality of the data distributions. We performed a non-paired test for the influence of the concept because the ’concept’ variable did not have a clear paired nature due to its computation (i.e., compliance is calculated for each participant and depends on the mean score obtained for each group). As post hoc tests, we used Mann–Whitney *U* tests to measure the significance of differences between concepts and between groups.

We measured intra-participant consistency by comparing test and retest trials. To do this, we calculated the proportion of duels of sounds with identical results both in test and retest trials. Because the retest scores were not normally distributed, we performed a Friedman test to test for the effect of concept and a Kruskal–Wallis test to test for the effect of group. Then, we performed post hoc Wilcoxon and Mann–Whitney *U* tests for the concept and the participant group.

**Figure 2:**
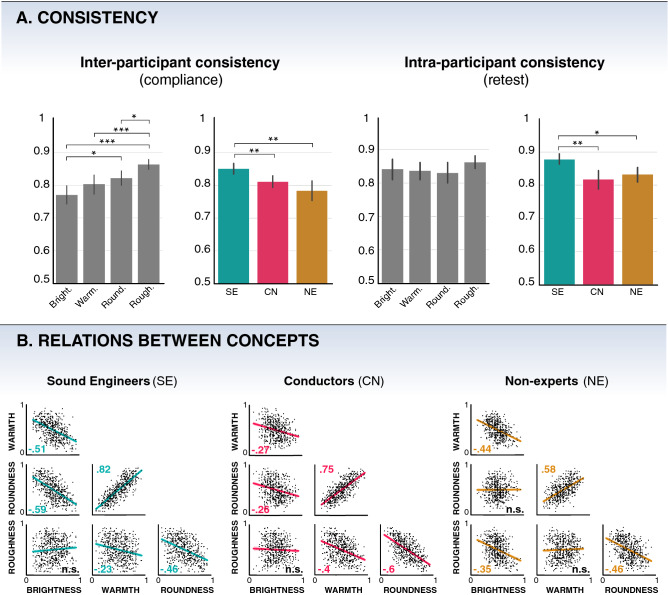
Behavioral results of the BWS experiment. (**A**) inter-participant (left) and intra-participant (right) consistency across concepts and populations (*p < .05; **p < .01; ***p < .001). (**B**) Correlations between BWS scores of each concept for each group of participants. SE: Sound Engineers (teal), CN: Conductors (red), NE: Non-Experts (yellow).

We computed Pearson’s correlation coefficients between sets of scores to compare results between concepts and populations. Additionally, we assessed statistical differences between all correlations with the Steiger test. See Fig. 2 for a schematic presentation of behavioral results.

Finally, we evaluated the differences in the frequency of use of the concepts by the expert participants (i.e., sound engineers and conductors) with a one-way ANOVA and post hoc t-tests.

We applied a Bonferroni correction to all post hoc tests to correct for multiple comparisons. All statistical analyses were performed in *Python 3.8* with the *Pingouin* library (https://pingouin-stats.org/#).

### Feature analysis

In this section, we detail the analyses we led to explain the BWS scores associated with each concept and each population (Fig. 1C,D). First, we trained a machine learning (ML) model on a regression task for predicting scores of brightness, roundness, warmth, and roughness based on static (i.e., collapsed over time) acoustic features. Second, we evaluated the contribution of all features to the BWS score of each sound with Explainable Artificial Intelligence (XAI)^36^—a process that aims to give sense to the learning/predicting process of an ML model.

We extracted spectral and spectro-temporal features (median value and interquartile ratio) with the *Librosa* library^37^, and temporal features with the Python version of the *timbre toolbox*^38^ (see Fig. 1C). We computed a Harmonic-to-noise ratio (HNR) metric with *Parselmouth*^39^. We also computed the Modulation Power Spectrum (MPS) roughness—a metric corresponding to the average energy present in the 30–150 Hz range on the time modulation axis of the modulation power spectrum^40^ (see Supplementary Information). We pruned the feature set down to 15 by performing a multicollinearity check and manually removing redundant features (see Supplementary Information). We included meta features associated with the instrumental specificities of each sound, i.e., the type of instrument and the playing technique with the one-hot encoding approach (i.e., either one or zero depending on the presence/absence of the property).

Next, we trained an ML model to predict the scores associated with a sound concept. For each concept and population, we performed a fivefold regression task using a tree-based model in the XGBoost gradient boosting framework^41^. The model would take the acoustic features and meta-features as input (X), and the BWS scores as output (y) for each concept and each population. We assessed the predictive accuracy of the model for each concept and population by computing the coefficient of determination (R^2^) between the model’s predictions on the test set and the actual score values (see Fig. 1D). We chose this model because it provided the highest R^2^ values compared to other models (multilinear regression, Lasso^42^, neural networks).

We measured the contributions of features for all concepts by computing their SHAP values. Conveniently, the SHAP library is a flexible XAI tool that provides a wrapper to explain any type of ML model and task^43^. For a given sound, the SHAP value of a feature is based on the computation of Shapley values^44^—a game theory tool that evaluates the marginal contribution of a feature to the output prediction of an item. SHAP values can be positive or negative. Thus, the explanation of the model strategy for predicting scores lies in the assignment of a SHAP value to each sound, hence enabling both global and local information on feature contributions. We used the *treeExplainer* function to evaluate the contribution of features to our prediction of BWS scores. Such a tool allowed us to explain any dependence of the concepts studied on the acoustic features, whether linear or not.

## Results

### Consistency across participant groups and concepts

Figure 2A reports the compliance (left) and retest (right) results across participant groups (SE: sound engineers; CN: Conductors; NE: non-experts) and concepts.

Our results show a main effect of concept on compliance scores (H(3) = 27.3, *p* < 0.001). Among all three groups, roughness (86%) was the most significantly consensual compared to the other concepts (U_rough./bright._ = 63.0, *p* < 0.001; U_rough./warm._ = 106.0, *p* < 0.001; U_rough./round._ = 148.5, *p* = 0.025). The second most consensual concept was roundness (82%), where participants showed significantly more consistency than for brightness (U_round./bright._ = 159.0, *p* = 0.048). The third most consensual concept was warmth (80%) and the least consensual was brightness (77%). There was no significant difference between brightness and warmth (U_bright./warm._ = 162.5, *p* = 0.427), nor between warmth and roundness (U_warm./round._ = 252.5, *p* = 1.0).

We observed a main effect of group on compliance score (H(2) = 15.2, *p* < 0.001). Specifically, sound engineers were significantly more consistent (85%) than the other groups of conductors (81%) and sound engineers (78%) (U_SE/CN_ = 748.0, p = 0.005; U_SE/NE_ = 772.0, *p* = 0.002). The difference of compliance between conductors and non-experts was not significant (U_CN/NE_ = 562.0, *p* = 1.0).

Regarding intra-participant consistency, we found a main effect of group on retests scores (H(2) = 12.6, *p* = 0.002). Once again, the sound engineer group showed a significantly higher intra-participant consistency (87%) compared to non-experts (83%) and conductors (82%) (U_SE/CN_ = 751.5, *p* = 0.004; U_SE/NE_ = 720.0, *p* = 0.015). There was no significant difference between conductors and non-experts (U_CN/NE_ = 456.0, *p* = 0.445), and there was no significant effect of concept (H(3) = 2.2, *p* = 0.534) on retests. We note that the high retests values for all participants attest for the absence of impact of fatigue on our results.

### Relations between BWS scores

We investigated the relationships between concepts by correlating the BWS scores associated with each concept between them (Fig. 2B).

For the three groups, brightness was negatively correlated to warmth (r_SE_(519) = − 0.51, p_SE_ < 0.001; r_CN_(519) = − 0.27, p_CN_ < 0.001; r_NE_(519) = − 0.44, p_NE_ < 0.001), roughness was negatively correlated to roundness (r_SE_(519) = − 0.46, p_SE_ < 0.001; r_CN_(519) = − 0.60, p_CN_ < 0.001; r_NE_(519) = − 0.46, p_NE_ < 0.001), and warmth was positively correlated to roundness (r_SE_(519) = 0.82, p_SE_ < 0.001; r_CN_(519) = 0.75, p_CN_ < 0.001; r_NE_(519) = 0.58, p_NE_ < 0.001).

Some relationships between concepts were exclusively shared between sound engineers and conductors. Hence, for the two experts population, we observed that roundness was negatively correlated to brightness (r_SE_(519) = − 0.59, p_SE_ < 0.001; r_CN_(519) = − 0.26, p_CN_ < 0.001), warmth was negatively correlated to roughness (r_SE_(519) = − 0.23, p_SE_ < 0.001; r_CN_(519) = − 0.40, p_CN_ < 0.001), and roughness and brightness were not significantly correlated. In contrast, for the non-expert group, brightness was negatively correlated to roughness (r_NE_(519) = 0.35, p_NE_ < 0.001), and the pairs brightness-roundness and warmth-roughness were not significantly correlated.

### Acoustic portraits of sound concepts

**Figure 3:**
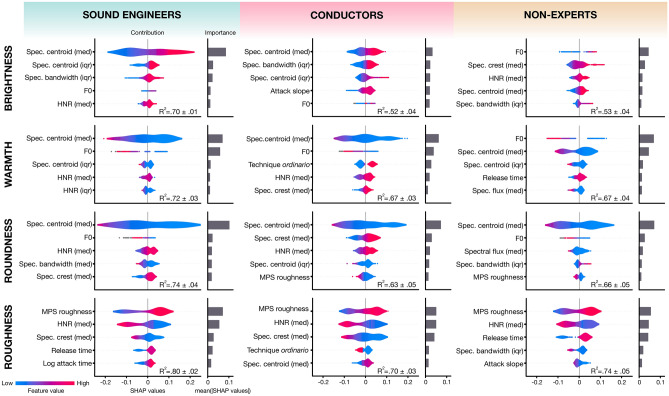
Top-5 features most explaining the regression model strategies for predicting the scores associated with sound concepts according to each group of participants. The figure represents both the nature of the contribution of each feature and its importance. The violin plots represent the contribution of each feature (SHAP Value on the x-axis) according to its value (hue color gradient). The thickness of the violin plot reflects the density of sounds for a feature value and contribution. The importance, i.e., the average of the absolute value of the contribution, is expressed in grey as a bar plot. med: median, iqr: interquartile range.

This section provides a description of the sound concepts for each group of participants, based on an ML-based analysis (see “Methods” section). Figure 3 reports the five most important features, along with the nature of their contribution, for the modeling of each concept according to the BWS scores of each population. The contribution of a feature is based on the averaged SHAP values computed on the test sets of the fivefold regression task. The mean accuracy of the model on the fivefold sets is reported with R^2^ values in Fig. 3.

The use of non-acoustic features such as source and playing mode did not drastically change the model results (∼ 0.02 on average compared to the presented scores). However, we kept them in the pool of features because of their positive, albeit small, impact on the prediction of each concept.

Overall, we found that roughness was the concept with the highest accuracy scores (R_SE_^2^ = 0.80; R_CN_^2^ = 0.70; R_NE_^2^ = 0.74), followed by roundness (R_SE_^2^ = 0.74; R_CN_^2^ = 0.63; R_NE_^2^ = 0.66) warmth (R_SE_^2^ = 0.72; R_CN_^2^ = 0.67; R_NE_^2^ = 0.67), and brightness (R_SE_^2^ = 0.70; R_CN_^2^ = 0.52; R_NE_^2^ = 0.53). Moreover, sound engineers' scores were predicted with more accuracy than the two other populations. Although some accuracy scores are low (e.g., R_CN_^2^ = 0.52 and R_CN_^2^ = 0.53 for brightness), previous studies have shown that the interpretability offered by the SHAP library and a model created via XGBoost remains valid even for low predictive accuracy^45^. Moreover, note that the importance of the contribution of these features remained mainly unchanged regardless of the dataset split performed before training.

Here, we present the features underlying the shared representation of the concepts according to all group's ratings. While roughness and roundness have similar top contributing features across groups, warmth, and above all, brightness show discrepancies. First, for all groups, we found that roughness depends mainly on noise components. Hence, roughness decreases with harmonic-to-noise ratio (HNR) and with Modulation Power Spectrum (MPS) roughness and spectral crest. Second, roundness ratings relied heavily on low spectral centroids, and to a lesser extent, on low fundamental frequencies (F0). Moreover, roundness is negatively impacted by noise components as shown by the contributions of HNR, spectral crest, and MPS roughness in the three groups acoustic portraits. Third, the results show that for all populations, warmth is strongly dependent on low F0 values, more so than roundness. In addition, according to expert groups, a warm sound should also not be too noisy (e.g., HNR and spectral crest), which is less relevant for non-experts. Fourth, sound engineers mainly related brightness to a high spectral centroid. The conductors also associated brightness mainly with spectral centroid, but the importance of its contribution is more shared with other features such as the spectral bandwidth, the attack slope, and the F0. Finally, according to non-experts’ results, brightness relies heavily on F0 and noise components. In other words, according to the non-expert group, a bright sound is roughly a high-pitched sound with low noise.

### Frequency of use of sound concepts

With no significant distinction between sound engineers and conductors, expert participants evaluated that they use roughness significantly less than brightness (t(15) = 5.4, *p* < 0.001) and roundness (t(15) = 5.2, *p* < 0.001).

## Discussion

In the present study, we investigated the mental representations of four sound concepts, namely, brightness, warmth, roundness, and roughness within groups of sound engineers, conductors, and non-expert participants. To do this, we used a dataset of orchestral sounds showcasing a great diversity of instrument timbres and playing techniques that participants rated on the four sound concepts using Best–Worst Scaling. To our knowledge, this investigation is the first to reveal and acoustically explain similarities and discrepancies in the mental representations of sound concepts between participant groups of different expertise based on acoustic portraits.

The results in terms of concept relations and acoustic portraits echo many findings of previous sound semantics research. First, we found that the spectral centroid is unanimously the principal feature of warmth and roundness^10,25^ and that expert participants, also associated it with brightness^10,14,16,24,25^. Second, we found that roughness strongly depends on noisiness and time-varying features^25,40^. Third, regarding relations between the sound concepts, most of our results (see Fig. 2B) are congruent with findings observed in the literature, such as the proximity of the concepts of warmth and roundness, their relative opposition to brightness^10,18^, the opposition of roundness and roughness^18^, as well as the absence of correlation between roughness and brightness^10,18^.

Thanks to the fine-grained acoustic descriptions obtained, we can unravel the specific representations of warmth and roundness. First, for all groups, the resemblance between roundness and warmth seems to be mostly explained by their dependency on low spectral centroid values. Second, one may notice that the two concepts differed in that a low pitch has more importance for warmth than roundness. Third, we note that sound engineers, conductors, and to a lesser extent, non-experts evaluated having few noisy components as more prominent for round sounds than for warm sounds. Moreover, we note the negative impact of the MPS roughness feature on roundness scores for conductors and non-experts. Finally, these observations corroborate the fact that participants evaluated roughness—which strongly depends on noise metrics (i.e., HNR, spectral crest, MPS roughness)—as being more negatively correlated to roundness than warmth.

According to sound engineers and conductors, they frequently use brightness for sound description, while they rarely use roughness. In contrast, our results show that roughness is the most consensual concept across groups, unlike brightness. Brightness has been generally associated with strong high-frequency components^16,18,24^ and high fundamental frequency^46,47^. While being faithful to these findings, our acoustic results and conceptual relationships account for discrepancies between groups in the mental representation of brightness. First, coherently with the aforementioned research, sound engineers mostly associated brightness with the median spectral centroid. This explains the nature of its relationship with roundness and warmth which have an inverse dependence on spectral centroid. Second, the conductors also associated brightness with spectral centroid, but its importance is more distributed with other features like the spread of spectral bandwidth, the attack slope, and the F0. This specificity explains the significantly lower correlation of brightness with roundness and warmth (Z_bright./warm._(519) = 4.39, p < 0.001; Z_bright./round._(519) = 5.92, p < 0.001; Steiger’s Z test) for the conductors compared to the sound engineers (see Fig. 2B). Third, in contrast with the experts, non-experts mainly associated brightness with the F0 and the quantity of noise (i.e., HNR and spectral crest). In other words, for the non-expert group, a bright sound is a high-pitched sound with low noise. This explains why, according to this group, brightness is opposed to warmth, which is also strongly related to F0, and to roughness, which is strongly dependent on noise features. This is also expressed in the measured correlations between scores (see Fig. 2). The negative correlation between warmth and brightness seems to be mainly based on opposite F0 dependencies, while the lack of correlation between roundness and brightness may stem from their common relationship to the amount of noise which is compensated by their opposite F0 dependencies.

Previous research has provided evidence of the superiority of sound and music experts when evaluating the acoustic aspects of sounds^11,24^. Going further, we investigated the influence of expertise in the vocabulary of sound professional communication. Specifically, we show through inter-participant consistency and acoustic explanations, that individuals with different sound expertise working together—like a sound engineer and a conductor in a mixing session, or a marketing representative and a sound designer—do not necessarily have the same fine understanding of well-known sound concepts. Thus, concepts like roundness and roughness are the most consensual whereas brightness and, to a lesser extent, warmth express specific understandings across participant groups (see Fig. 2A). Moreover, according to consistency results, sound engineers provide greater agreement than other groups for the understanding of sound concepts. Incidentally, we found a correlation (r(11) = 0.89, *p* < 0.001) between inter-participant consistency and the accuracy of the models (R^2^) for each group and each concept. The performance of the model thus seems to depend strongly on the consistency within groups rather than on the nature of the acoustic features.

Current views on sound semantics aim to make sense of the mechanisms involved in the pairing of a metaphorical sound concept with its source domain (e.g., touch for warmth or roughness) from the perspective of crossmodal correspondences^46,48–50^. Our results do not give any indication of the actual sensory coupling that might underlie the mental representations of these sound concepts. Nonetheless, we wish to question the immutability of the four concepts' shared mental representation in expert communities that use this type of sensory metaphor in professional settings. While roughness is the least used concept, it is the most consensual, and its acoustic representation is very stable across participant groups (see Fig. 2). This suggests that, despite any sound or music education, the common metaphorical use of roughness remains unchanged. In contrast, our findings regarding brightness—a key term in expert sound communication—seem to express a certain diversity in the shared mental representations for each group, both through consistency scores and acoustic analyses. This result may indicate that the meaning of brightness got specified through its use in a professional context or through the sound education of expert participants. The specificity of brightness is such, that even between two groups of experts, the concept has different levels of complexity (see Fig. 3). Although the explanation for such a phenomenon remains to be thoroughly explored, our results suggest that brightness is reminiscent of the concept of dead metaphor^51^. A dead metaphor is a figure of speech derived from the repeated verbal use of a metaphor in a specific community. Thus, a term originally metaphorical (i.e., using a term coming from a source domain in a distinct target domain) becomes a term endogenous to the discourse attached to the domain of interest, here the sound domain. Thus, the meaning of brightness, unlike roundness, which is also widely used but shared across populations, has evolved with the expertise of our participants. In the end, sound and music professionals interact in partially independent discursive domains, making possible processes of individuation of linguistic uses such as the metaphorical description of sound.

One limitation of the current study is the low sample size of each socio-professional category (N = 8), which restricts, for example, our ability to investigate gender differences in the data. However, it is important to note that the BWS method we used is different from other studies using traditional inferential statistical methods. Indeed, the BWS algorithm computes a ranking of the dataset for each attribute and group, which requires an optimal number of annotations for convergence. Based on previous research^29^, a sample size of eight is an optimal trade-off between data collected and sorting algorithm precision. Hence, despite the low sample size, our results showed high consistency within groups (see “Behavioral results”) and stable weights of acoustic features across different dataset splits during the acoustic analyses. Nonetheless, future studies should consider increasing sample sizes and investigate the question of power in BWS designs further.

## Conclusion

With this work, we assessed the impact of sound expertise of three groups of participants on their mental representations of metaphorical sound concepts. To do so, we acoustically explained brightness, roundness, warmth, and roughness according to the evaluation of a sound dataset on these terms by sound engineers, conductors, and non-experts of sound. Surprisingly, the term most used in the expert domains (brightness) is much less consensual than the least used term (roughness). Furthermore, we went deep into the acoustic descriptions of the concepts revealing the existing relationships between concepts according to the ratings of each group of participants. For example, we studied the subtle specifics of roundness and warmth, which are spectrally very similar, for all participants, but also for each group. With this work, we bring a fine understanding of the technical vocabulary of sound, as well as an ergonomic methodology based on Best–Worst Scaling that can be applied in the future in crowdsourcing contexts, paving the way for the study of other complex sound concepts (e.g., richness, fullness) as perceived by other populations (e.g., brass instrument player vs string instrument player), but also on other issues (e.g., voice identity, sound dataset validation).

## Supplementary Information

Supplementary Information.

## Data Availability

Supplementary information and datasets generated and/or analyzed during the current study are available at https://osf.io/pxjw2/?view_only=539826a5d9eb4a3fb2ea922777e74bd3.
