# Supplementary material for: Shared mental representations underlie metaphorical sound concepts
Source: Sci Rep. 2023 Mar 30;13:5180. doi: 10.1038/s41598-023-32214-2 (PMC10063581; doi:10.1038/s41598-023-32214-2)
Supplement: Supplementary file 1 — Supplementary Information. [file 41598_2023_32214_MOESM1_ESM.pdf]

Supplementary information

Shared Mental Representations Underlie Metaphorical Sound Concepts.

Victor Rosi, Pablo Arias, Olivier Houix, Nicolas Misdariis & Patrick Susini

Supplementary Tables

Table S1. Related to data analysis. List of instruments per instrument families in the dataset.

Instrument family	Instruments
Strings	Violin<br>Alto<br>Cello<br>Doublebass
Woodwinds	Flute<br>Alto flute<br>Piccolo flute<br>Oboe<br>English horn<br>Clarinet<br>Bass clarinet<br>Bassoon<br>Contrabassoon<br>Alto saxophone
Brass	Trumpet<br>Trombone<br>French horn<br>Tuba
Mallets	Glokenspiel<br>Xylophone<br>Vibraphone<br>Marimba
Others	Harpa<br>Guitar<br>Accordion

Table S2. Related to data analysis. List of playing techniques per instrument families in the dataset. Instruments with specific playing techniques are presented in parenthesis.

Instrument Family	Playing technique
All	Ordinario*
Strings	Non vibrato<br>Pizzicato<br>Pizzicato Bartók<br>Sul ponticello<br>Artificial harmonics
Winds	Flatterzunge<br>Staccato<br>Multiphonics (woodwinds)<br>Semi-aeolian (flute/clarinet)<br>Brassy (brass)<br>Straight/cup/harmon mute (brass)<br>Play & Sing (tuba)<br>Pedal tone (trombone)
Mallets	Soft sticks<br>Hard sticks<br>Arco
Plucked strings	Harmonics

Table S3. Related to Feature analysis. List of acoustic features used in the. In bold: selected features for the acoustic analysis after multicollinearity check. *: computed with librosa[1]; (**) computed with the timbre toolbox[2]; ***: computed with Parselmouth[3]. med: median; iqr: interquartile range.

**Spectral centroid (med, iqr)***
**Spetral bandwidth (med, iqr)***
Spectral contrast (med, iqr)*
**Spectral crest (med, iqr)***
Spectral flatness (med, iqr)*
Spectral rolloff (med, iqr)*
**Spectral flux (med, iqr)***
Zero-crossing rate (med, iqr)*
**Log-attack time****
**Attack slope****
Decrease slope**
**Release time****
**Harmonic-to-noise ration (med, iqr)*****
**Fundamental frequency**
**MPS roughness**

Supplementary note on *Modulation Power Spectrum (MPS) Roughness*

Here is a presentation of the roughness metric introduced in the results of the paper.

The **Modulation Power Spectrum** (MPS) is the 2D-Fourier transform of the spectrogram of a sound. It is often used to characterize and represent the spectro-temporal specificities of sounds[4,5]. It depicts the power of modulation on both temporal and spectral dimensions.

On the MPS, the region between 30 and 150 Hz on the temporal modulation axis has been proven to be a good predictor of psychoacoustical roughness[4].

Figure S1 reports on two MPSs of saxophone sounds playing the same note (C4). The one on the left is an *ordinario* sound played pianissimo with a generally low roughness rank, the one on the right is a multiphonic with a generally high roughness rank. The green rectangles indicate the region of interest including the rough content of sounds. On the multiphonic MPS (right panel of Fig. S1.A), we clearly see modulation components (i.e., vertical lines) that contributes to the roughness of the sound. The MPS roughness metric introduced in this paper is the mean energy within the frequency range 30-150Hz. The MPS roughness values in the dataset range from 31.0 to 70.9 (arb. unit). Here, it equals 49.6 for the *ordinario* sound, and 60.9 for the multiphonic sound.

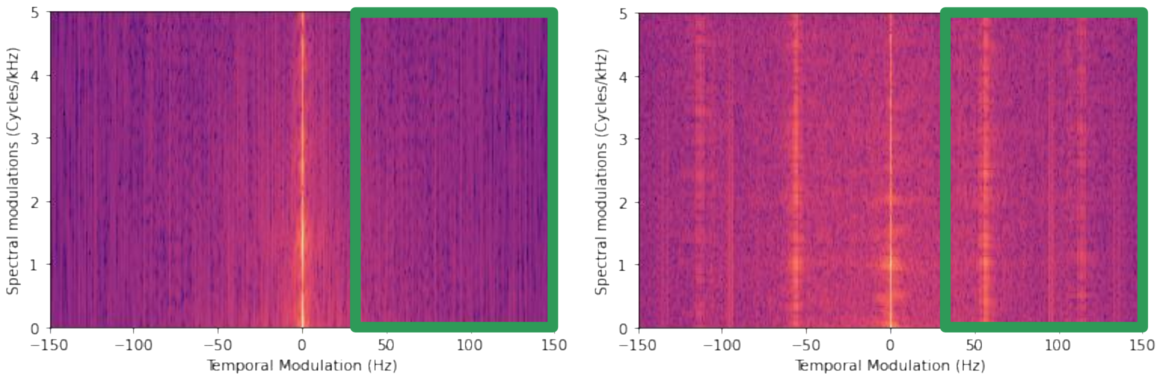

Figure S1. Modulation power spectrums (MPS) of an ordinario saxophone sound (left) and a saxophone's multiphonic (right). The green frame indicates the region capturing the roughness of a sound.

As an example of the information carried by this feature in our results, Figure S2 presents the correlation of brightness and roughness scores according to sound engineers judgments with the roughness metric.

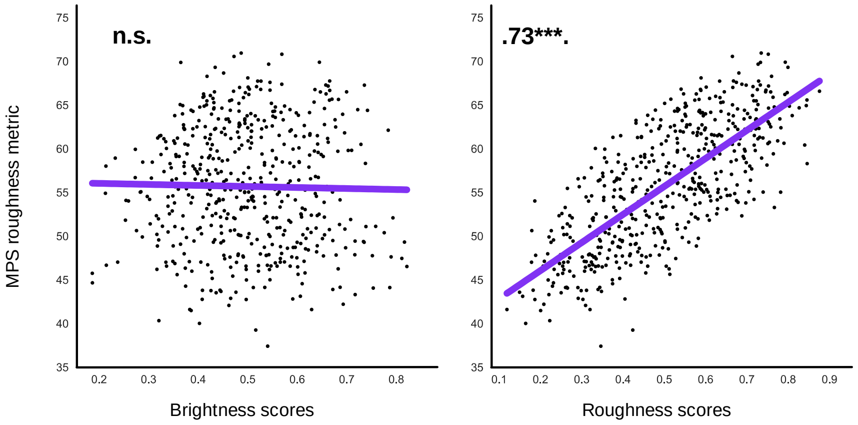

Figure S2. Correlations of MPS with the brightness and roughness scores provided according to sound engineers judgments.

Supplementary References

1. McFee, B. *et al.* librosa: Audio and music signal analysis in python. in *Proceedings of the 14th python in science*

conference vol. 8 18–25 (Citeseer, 2015).
2. Peeters, G., Giordano, B. L., Susini, P., Misdariis, N. & McAdams, S. The timbre toolbox: Extracting audio descriptors from musical signals. *J. Acoust. Soc. Am.* **130**, 2902–2916 (2011).
3. Jadoul, Y., Thompson, B. & de Boer, B. Introducing Parselmouth: A Python interface to Praat. *J. Phon.* **71**, 1–15 (2018).
4. Arnal, L. H., Flinker, A., Kleinschmidt, A., Giraud, A.-L. & Poeppel, D. Human screams occupy a privileged niche in the communication soundscape. *Curr. Biol.* **25**, 2051–2056 (2015).
5. Thoret, E., Caramiaux, B., Depalle, P. & Mcadams, S. Learning metrics on spectrotemporal modulations reveals the perception of musical instrument timbre. *Nat. Hum. Behav.* **5**, 369–377 (2021).
